# Transplant ureter should be stented routinely

**DOI:** 10.4103/0970-1591.70594

**Published:** 2010

**Authors:** Ritesh Mongha, Anant Kumar

**Affiliations:** Departments of Urology and Kidney Transplant, Fortis Hospital, Vasant Kunj, New Delhi-110 070, India

**Keywords:** Stent, transplant ureter

## Abstract

Vesicoureteric complications present early after transplantation and contribute to patient morbidity, graft loss and mortality. Ureteral stenting provides a decrease in ureteroneocystostomy anastomotic complications following renal transplantation. There should be prophylactic stent insertion with endoscopic removal at a designated time post transplantation. With the addition of antibiotic prophylaxis post transplantation, ureteric stenting does not increase the rate of urinary tact infections. There is no significant increase in cost for stenting during transplantation in comparison to management of major ureteric complications. Routine stenting causes significant cost-saving per year and prevents anastomotic complications. It is wise to stent the transplant ureter routinely.

## INTRODUCTION

Kidney transplantation is the treatment of choice for end-stage kidney disease. Ureteroneocystostomy anastomotic leakage and/or strictures complicate 3–9% of all renal transplants.[1–3] These urinary complications remain the most common technical complication associated with contemporary renal transplantation.[2–4] Numerous studies have addressed the issue of routine anastomotic stenting in renal transplantation in an attempt to decrease the rate of urinary complications but the debate continues.[5–13] Many selectively stent only difficult anastomosis or in circumstances where the vesicoureteric viability may be additionally compromised. Vesicoureteric complications present early after transplantation and contribute to patient morbidity, graft loss and mortality. It has been our policy to use stents routinely in all cases.

We would like to address the following three categories:

## PREVENTION OF MAJOR URETERIC COMPLICATIONS—PREVENTION IS BETTER THAN CURE!

Initially, ureterovesical anastomosis was done using a transvesical Leadbetter-Politano approach which is presently superceded by the extravesical ureteroneocystostomy (Lich-Gregoir) approach.[14–16] Refinement in surgical techniques and the introduction of new immunosuppressive protocols resulted in a significantly decreased incidence of urological complications from around 20% in the 1970s to less then 5% in the 1990s.[17–20] Vesicoureteric complications present either as urine leaks, ureteric stenosis or obstruction (major urological complications (MUC)). In the absence of technical complications, ureteric ischemia is thought to be chiefly responsible for the early ureteric complications post transplantation.[21] Minor ureteric leak and obstruction have been successfully treated with “double-J” stent insertion, prompting surgeons to contemplate its use as a prophylactic measure in transplantation and other urological procedures.[22] Major urological complications (MUCs) mostly originate from the vesicoureteric anastomosis, present early after transplantation (within three months),[23] and could contribute to patient morbidity, graft loss and mortality.[24] Patients with urinary anastomotic complications have significantly longer hospitalizations in the first year of transplantation. The readmissions suggest that patient-specific morbidity is directly related to the anastomotic complication and graft dysfunction. Fluid balance abnormalities may develop which manifest as fluid overload and increased acute cardiac events. Urinary tract (UT) and non-UT infectious complications are also significantly increased in this patient population. Acute renal failure is almost 2.5 times more likely to develop in patients with urinary anastomotic complications. The increased patient morbidity associated with urinary anastomotic complications translates into increased costs.

Conventional native ureteric repairs over stents are widely accepted to have a better outcome.[25] In addition they have been successfully used in pyeloplasty, ureterovesical reconstruction and in the management of stone disease.[26,27] There are many theoretical benefits of prophylactic stenting. A stent has been reported to make the anastomosis technically easier to perform and the final luminal diameter may be larger.[8] A stent probably avoids ureteral bending, kinking or external compression from perigraft fluid collections. Stenting may eliminate compression from a tight submucosal tunnel and equalize ureteral and bladder pressure, facilitating urine drainage during the high diuresis experienced in the early post-transplantation period. Finally, the stent physically traverses the anastomosis, preventing urinary extravasations though potential gaps in the suture line or small areas of necrosis and effectively decreasing the risk of urinary complications. Moreover, prophylactic stenting can treat minor leaks and obstruction at the anastomotic site. Routine stenting was clinically demonstrated to improve renal function in the early postoperative period in a prospective, randomized study.[6] Perigraft fluid collections were also shown to be significantly decreased with stenting;[8] similarly the drain output is significantly less when intraoperative drains are routinely placed around the transplant graft.[5]

In all randomized and quasi-randomized controlled trials looking at the use of double-J stents to prevent urological complications, the incidence of MUCs ranged between 0 and 5% in stented patients (median 1.0%) and between 0 and 17.3% (median 7.0 %) in the non-stented patients [Table 1].[5–11] A three-phase longitudinal study done in the year 2000 included 670 consecutive living related renal transplants. In Phase 1, a stent was introduced as and when required. Only 15 of 170 patients were stented. In Phase 2, 57 and 43 cases were randomized to stenting and no stenting, respectively. The stent was removed after four weeks. In Phase 3, all patients received a stent, which was removed 10 to 14 days just before discharge. In Phase 1 the major ureteral complication rate was 8.8%, which decreased to 3% in Phase 2 when half of the cases were stented. In Phase 3 there was only one ureteral complication (0.04%) in 400 patients, of whom all received a stent. The overall ureteral complication rate in non-stented and stented cases was 8.5% (18 of 213) and 0.22% (1 of 457) respectively.[23]

**Table 1:** Major urological complications

Study	Complications (stent)	Patients (stent)	Incidence (stent)	Complications (no stent)	Patients (no stent)	Incidence (no stent)
Bassiri 1995[5]	0	35	0%	3	37	8.1%
Benoit 1996[6]	1	97	1%	10	97	10.30%
Dominguez 2000[7]	5	143	3.5%	9	137	6.6%
Guleria 1998[9]	1	54	1.9%	3	54	5.6%
Kumar 1998[8]	0	57	0%	3	43	7%
Osman 2004[11]	2	50	4%	0	50	0%
Pleass 1995[10]	0	150	0%	26	150	17.3%

## COMPLICATIONS OF STENTING

The most significant theoretical complication in the use of a stent is an increase in the number and severity of urinary tract infections (UTIs). Other possible complications include persistent hematuria, bladder discomfort, stent migration, breakage, encrustation and complications during removal. Most centers have adopted a policy of prophylactic stent insertion with endoscopic removal at a designated time post transplantation in an effort to reduce the rate of MUCs.[28] UTIs, in general, were more common in stented patients unless the patients were prescribed cotrimoxazole in which case the incidence was equivalent. Stents appear to be generally well tolerated, although studies using longer stents (20 cm) for longer periods (> six weeks) had more problems with encrustation and migration. These possible complications can be avoided by using the stents for the minimal possible duration. The optimal duration of stenting in renal transplantation is not yet established. In a case-controlled study, it was found that stenting for two weeks avoids complications of prolonged use of stents without compromising the benefits.[29] In a similar study it was suggested that the routine use of a double-J stent for ureterovesical anastomosis neither significantly increased UTI rates, nor decreased the incidence of urinary leaks, but may decrease the gravity of the latter as evidenced by the need for surgical intervention.[30]

The maximum reported non-infectious complications were irritative symptoms 5.6%,[9] breakage 2.0%,[6] migration/malposition/expulsion 7.4%,[9] encrustation/urolithiasis 5.7%[5] and “forgotten” stents 7%[8] [Table 2]. Earlier removal of stent at two weeks does not increase morbidity (rate of urological complications) in transplant recipients and prevents stent-related complications associated with prolonged use of stent. It obviates the risk of forgotten stents as well as curtails the cost of second admission for stent removal.[29] There is no evidence that the presence of a stent predisposes to recurrent or severe hematuria.

**Table 2:** Non-infectious complications

Study	Irritative symptoms	Breakage	Migration/malpositioning	Encrustation	Forgotten	Expulsion
Bassiri 1995[5]	NR	NR	NR	5.7%	NR	0
Benoit 1996[6]	NR	2.1%	1.0%	2.1%	NR	1.0%
Dominguez 2000[7]	0%	0%	0%	0%	NR	0%
Guleria 1998[9]	5.6%	0 %	7.4%	0 %	NR	7.4%
Kumar 1998[8]	5.3%	0 %	0%	0 %	7.0 %	0
Osman 2004[11]	NR	0 %	4.0%	0 %	0	0
Pleass 1995[10]	NR	0 %	“+”	“++”	NR	> 1 patient

In another study it has been suggested that ureteral stasis may cause tubuloepithelial injury and slow down the decrease in creatinine levels. They concluded that Double J Stent (DJS) did not increase UTIs but provided a smooth decline in creatinine levels, which may reduce the question of acute rejection.[31]

## INPATIENT HOSPITAL COST OF MAJOR UROLOGICAL COMPLICATIONS VS ROUTINE STENTING

The incremental inpatient hospital costs associated with a urinary complication during the first 12 months following renal transplantation were 145% of the cost of renal transplantation without this complication. Notably, this value does not include inpatient hospital indirect costs, any expenses generated on an outpatient basis or elsewhere, or any inpatient or outpatient professional costs. Thus, the actual incremental cost associated with a urinary anastomotic complication following renal transplantation is significantly higher than this value.[32] There is no significant increase in cost for stenting during transplantation except the cost of stent which costs a few hundred rupees and can be electively removed at the time of discharge avoiding second admission. This causes significant cost saving per year and prevents anastomotic complications and avoids the morbidity of prolonged stenting.

## META-ANALYSIS

A recent meta-analysis evaluated five prospective, randomized, controlled clinical trials of routine stenting following renal transplantation and indicated that the collective urinary complication rate following routine stenting was 1.5% compared to 9% without stenting (OR 0.24, *P* <0.0001).[1] The OR for urinary complications with routine stenting varied among these five prospective studies at between 0.02 and 0.53 with three of the five demonstrating statistical significance independently.[5–9] Similarly, a Cochrane review evaluated these five series and included two additional prospective, randomized series. The study concluded that the collective urinary complication rate following routine stenting was 1.0% compared to 7.0% without stenting (OR 0.24, *P* = 0.02).[12]

## CONCLUSION

The review of the literature appears to tilt the balance heavily in favor of routine prophylactic stenting in renal transplant recipients. Transplant units currently using antibiotic regime as prophylaxis for pneumocystis carnii should not notice an excess of stent-related infections. The use of an appropriate size of stent and early removal at two weeks prevents morbidity and stent-related complications. It is wise and cost-effective to stent the ureteroneocystostomy after transplantation.
